# Antibiotic resistance and extended-spectrum β-lactamase in *Escherichia coli* isolates from imported 1-day-old chicks, ducklings, and turkey poults

**DOI:** 10.14202/vetworld.2020.1037-1044

**Published:** 2020-06-10

**Authors:** Mona A. A. AbdelRahman, Heba Roshdy, Abdelhafez H. Samir, Engy A. Hamed

**Affiliations:** 1 Department of Bacteriology, Reference Laboratory for Veterinary Quality Control on Poultry Production, Animal Health Research Institute, Agriculture Research Center, P.O. Box 264, Dokki, Giza 12618, Egypt; 2 Department of Biotechnology, Reference Laboratory for Veterinary Quality Control on Poultry Production, Animal Health Research Institute, Agriculture Research Center, P.O. Box 264, Dokki, Giza 12618, Egypt

**Keywords:** *Escherichia coli*, extended-spectrum β-lactamase, imported, multidrug resistance, poultry

## Abstract

**Aim:**

Antimicrobial resistance is a global health threat. This study investigated the prevalence of *Escherichia coli* in imported 1-day-old chicks, ducklings, and turkey poults.

**Materials and Methods:**

The liver, heart, lungs, and yolk sacs of 148 imported batches of 1-day-old flocks (chicks, 45; ducklings, 63; and turkey poults, 40) were bacteriologically examined for the presence of *E. coli*.

**Results:**

We isolated 38 *E. coli* strains from 13.5%, 6.7%, and 5.4% of imported batches of 1-day-old chicks, ducklings, and turkey poults, respectively. They were serotyped as O91, O125, O145, O78, O44, O36, O169, O124, O15, O26, and untyped in the imported chicks; O91, O119, O145, O15, O169, and untyped in the imported ducklings; and O78, O28, O29, O168, O125, O158, and O115 in the imported turkey poults. The *E. coli* isolates were investigated for antibiotic resistance against 16 antibiotics using the disk diffusion method and were found resistant to cefotaxime (60.5%), nalidixic acid (44.7%), tetracycline (44.7%), and trimethoprim-sulfamethoxazole (42.1%). The distribution of extended-spectrum β-lactamase (ESBL) and *ampC* β-lactamase genes was *bla*_TEM_ (52.6%), *bla*_SHV_ (28.9%), *bla*_CTX-M_ (39.5%), *bla*_OXA-1_ (13.1%), and *ampC* (28.9%).

**Conclusion:**

Imported 1-day-old poultry flocks may be a potential source for the dissemination of antibiotic-resistant *E. coli* and the ESBL genes in poultry production.

## Introduction

The global spread of antibiotic-resistant ­bacteria poses a potential threat to public health. The most important type of antibiotic resistance is that to β-lactamases, which has emerged as the result of the production of antibiotics containing β-lactamase-hydrolyzing enzymes because of the massive use of penicillins, cephalosporins, and carbapenems [1]. The global spread of extended-spectrum β-lactamase (ESBL) genes plays an essential role in the development of antibiotic resistance, which can be transmitted to humans through the consumption of animals for food, such as poultry, or by direct contact with contaminated poultry and their byproducts [2]. There are multiple routes of transmission of antibiotic-resistant bacteria, including plasmids, whole bacterial transmission, or mobile genetic element-mediated transmission [2,3]. The prolonged, uncontrolled use of b-lactam ­antimicrobials to treat many bacterial infections caused by members of the Enterobacteriaceae family, such as *Escherichia coli*, has resulted in the development of ESBL genes. Consequently, the number of β-lactamase-producing bacteria has increased [4]. An increase in the percentage of β-lactamase-producing *E. coli* has been observed among humans and in food samples, which are a potential serious risk to public health because of the considerable number of multidrug-resistant (MDR) genes [5].

*E. coli* is a Gram-negative bacterium that is a commensal in the gastrointestinal tracts of humans and animals, contributing to the development of antimicrobial resistance in commensal gut flora, which has an effect on the selection of antimicrobial agents as well as the spread of antimicrobial resistance [6]. *E. coli* is also important pathogen that causes diarrhea and death among humans and animals, and its presence gives an indication of the environmental status in poultry farms [7]. Moreover, it causes diseases in poultry, such as septicemia, swollen head syndrome, umbilical cord inflammation, egg yolk peritonitis, and chronic respiratory disease, resulting in reduced egg production and carcass condemnation, leading to drastic economic losses [7,8]. ESBLs hydrolyze the b-lactam ring in b-lactam antibiotics, giving rise to resistance to most b-lactam antibiotics, such as penicillins, cephalosporins, and the monobactam aztreonam. In addition, ESBL-producing Enterobacteriaceae have shown resistance to other antibiotic families, such as fluoroquinolones, ­trimethoprim-sulfamethoxazole (SXT), aminoglycosides, and tetracyclines, resulting in inadequate treatment [9-11]. TEM, SHV, and CTX-M are important families of ESBL enzymes that can destroy first-, second-, and third-generation cephalosporins, penicillin, and aztreonam. The β-lactamase inhibitors clavulanic acid and sulbactam can hinder the action of ESBL enzymes. *ampC* β-lactamases degrade first-, second-, and third-generation cephalosporins, penicillin, and aztreonam and are not suppressed by clavulanic acid or other β-lactamase inhibitors [12,13]. OXA is a plasmid-mediated β-lactamase that belongs to class D carbapenemases [13].

The study aimed to detect the prevalence of *E. coli* in imported chicks, ducklings, and turkey poults and to determine the prevalence ESBL and ampC genes.

## Materials and Methods

### Ethical approval

The study procedure was approved by the Reference Laboratory for Veterinary Quality Control on Poultry Production, Animal Health Research Institute, Egypt.

### Samples

We tested 15 samples each from 148 batches (45 chick, 63duckling, and 40 turkey poults) of imported 1-day-old flocks that had been quarantined for the detection of epizootic disease before entry into Egypt.

Bacteriological examination was conducted when the chicks, ducklings, and turkey poults were 15 days old. The parenchymatous organs (i.e., liver, heart, lungs, and yolk sacs) were pooled as previously described [14] (four pools of 15 livers, 15 hearts, 15 lungs, and 15 yolk sacs, respectively, per batch). Each pool was used for *E. coli* isolation and subsequent antimicrobial susceptibility testing. The total number of examined samples is shown in Table-1.

**Table-1:** Samples from examined imported flocks.

*Poultry* spp.	No. of examined batches	No. of subsamples	Total
One-day-old chicks	45	4	180
One-day-old ducklings	63	4	252
One-day-old turkey poults	40	4	160
Number of tested batches	148		Total no. of samples 592

### Isolation and identification of E. coli

*E. coli* isolation and serotyping were performed as previously described [15,16]. On the day of arrival from the quarantine to the laboratory, the samples were enriched in Buffered Peptone Water at 37 °C for 24 h and then samples were streaked on MacConkey agar (Oxoid Ltd., Basingstoke, UK) and incubated aerobically at 37°C for 24 h. Lactose-fermenting colonies were then picked and re-streaked on eosin methylene blue agar (Oxoid Ltd.) and incubated for 24 h at 37°C. Colonies with a green, metallic sheen were considered *E. coli*. These colonies were further biochemically tested for growth on triple sugar iron agar and lysine iron agar as well as for citrate utilization, urease production, and indole fermentation.

### Determination of antimicrobial sensitivity

The antimicrobial sensitivity was assessed according to the Clinical and Laboratory Standards Institute (CLSI) guidelines [17] using the Kirby–Bauer disk diffusion method on Mueller-Hinton agar plates (Oxoid) against 16 antibiotics (Oxoid), namely, penicillin G (10 U), amoxicillin + clavulanic acid (10-20 μg), cefotaxime (30 μg), imipenem (10 mg), SXT (1.25-23.75 μg), streptomycin (10 μg), gentamycin (10 μg), doxycycline (30 μg), tetracycline (30 μg), norfloxacin (10 μg), levofloxacin (5 μg), ciprofloxacin (5 μg), nalidixic acid (30 μg), chloramphenicol (30 μg), erythromycin (15 μg), and nitrofurantoin (300 μg).

The susceptibility of *E. coli* isolates to individual antimicrobial agents was determined and interpreted the following aerobic incubation at 37°C for 18-24 h, according to CLSI guidelines. Test results were considered valid if the diameters of the inhibition zones of the control *E. coli* strain (ATCC 25922) were within the performance ranges.

### Detection of ESBL and ampC genes for E. coli

DNA was extracted from the samples using aQIAamp DNA Mini Kit (Qiagen, Hilden, Germany) with modifications to the manufacturer’s recommendations. Briefly, 200 μl of the sample suspension was incubated with 10 μl of proteinase K and 200 μl of lysis buffer at 56 L for 10 min thereafter, 200 μl of 100% ethanol was added to the lysate. The sample was then washed and centrifuged according to the manufacturer’s instructions. The extracted DNA was eluted with 100 μl of elution buffer.

We performed polymerase chain reaction (PCR) on the extracted DNA samples. The primers (Metabion, Planegg-Martinsried, Germany) are detailed in Table-2 [18-20]. PCR was performed using an Applied Biosystems 2720 Thermal Cycler (Foster City, CA, USA) in a final reaction volume of 25 μl containing 12.5 μl of Emerald Amp MAX PCR Master Mix (Takara, Shiga, Japan), 1 μl of each primer (20 pM), 4.5 μl of H_2_O, and 6 μl of DNA template. The PCR products (20 μg/lane) were separated by electrophoresis on 1.5% agarose gel (Applichem GmbH, Darmstadt, Germany) in 1 ×Tris-borate-EDTA buffer at room temperature using a gradient of 5 V/cm. A. 100-1000-bp DNA ladder (GenedireX Inc., Flint Place Poway, CA, USA) was used to determine the fragment sizes. The gel was photographed by an Alpha Innotech gel documentation system (Biometra GmbH, Göttingen, Germany), and the data were analyzed using the associated system software.

**Table-2:** Primers sequences, target genes, amplicon sizes, and cycling conditions.

Target gene	Primers sequences	Amplified segment (bp)	Primary denaturation	Amplification (35 cycles)			Final extension	Reference

				Secondary denaturation	Annealing	Extension		
*bla*_TEM_	ATCAGCAATAAACCAGC	516	94°C 5 min	94°C 30 sec	54°C 40 sec	72°C 45 sec	72°C 10 min	[18]
	CCCCGAAGAACGTTTTC							
*bla*_SHV_	AGGATTGACTGCCTTTTTG	392	94°C 5 min	94°C 30 sec	54°C 40 sec	72°C 40 sec	72°C 10 min	
	ATTTGCTGATTTCGCTCG							
*bla*_OXA-1_	TCAACTTTCAAGATCGCA	609	94°C 5 min	94°C 30 sec	54°C 40 sec	72°C 45 sec	72°C 10 min	
	GTGTGTTTAGAATGGTGA							
*ampC*	TTCTATCAAMACTGGCARCC	550	94°C 5 min	94°C 30 sec	50°C 40 sec	72°C 45 sec	72°C 10 min	[19]
	CCYTTTTATGTACCCAYGA							
*bla*_CTX-M_	ATG TGC AGY ACC AGT AAR GTK ATG GC TGG GTR AAR TAR GTS ACC AGA AYC AGC GG	593	94°C 5 min	94°C 30 sec	54°C 40 sec	72°C 45 sec	72°C 10 min	[20]

## Results

### E. coli isolation

*E. coli* infections in the internal organs of apparently healthy 1-day-old imported batches of flocks presented as heart and lung congestion, pneumonia, pericarditis, perihepatitis, and omphalitis on postmortem examination. We detected *E. coli* in 25.6% (38/148) of the total examined samples. The recovery rates after *E. coli* isolation from the imported chicks, ducklings, and turkey poult samples were 13.5%, 6.7%, and 5.4%, respectively (Table-3).

**Table-3:** Incidence of *E. coli* was isolated from imported flocks as follow.

Flock type	No. of examined flock batches	No. of isolated *E. coli* from each batch	Incidence of *E. coli/* each flock batches	Incidence of *E. coli*/total flock batches	Serogroups
Imported chicks	45	20	44.4% (20/45)	13.5% (20/148)	O91, O125, O145, O78, O44, O36, O169, O124, O15, O26, (5) untyped isolates
Imported ducklings	63	10	15.8% (10/63)	6.7% (10/148)	O91, O119, O145, O15, O169, (1) untyped isolate.
Imported turkey poults	40	8	20% (8/40)	5.4% (8/148)	O78, O28, O29, O168, O125, O158, O115
Total	148	38		25.6% (38/148)	

E. coli=Escherichia coli

### Serotyping of E. coli isolates

We identified 17 serotypes in the *E. coli* isolates, of which the most common were O91, O125, O145, O78, O169, O15, and untyped isolates. The untyped isolates were unable to be typed using the antisera available in Egypt. The serotype distribution was O91, O125, O145, O78, O44, O36, O169, O124, O15, O26, and untyped in the imported chicks; O91, O119, O145, O15, O169, and untyped in the imported ducklings; and O78, O28, O29, O168, O125, O158, and O115 in the imported turkey poults.

### Antimicrobial sensitivity of isolated E. coli strains

The highest rate of resistance was found to cefotaxime (60.5%), tetracycline (44.7%), nalidixic acid (44.7%), and SXT (42.1%). A moderate resistance rate was shown to streptomycin (36.8%), doxycycline (26.3%), ciprofloxacin (26.3%), and norfloxacin (21%). The *E. coli* isolates were most susceptible to gentamycin (18.4%), levofloxacin (15.8%), nitrofurantoin (12.5%), chloramphenicol (12.5%), amoxicillin-clavulanic acid (5.2%), and imipenem (2.6%) (Tables-4 and 5). Of the *E. coli* isolates, 22/38 (57.8%) were MDR because they showed resistance to three or more classes of antimicrobial agents, excluding erythromycin and penicillin (Table-5).

**Table-4:** Antibiotic susceptibility of 38 *E. coli* isolates from imported flocks using disk diffusion.

Antimicrobial class	Antimicrobial agent	Sensitive rate (%)	Intermediate sensitive (%)	Resistant rate (%)
β-lactams	Penicillin	0	0	100
β-lactam/β-lactamase inhibitor	Amoxicillin-clavulanic acid	79	15.8	5.2
β-lactam (cephalosporin)	Cefotaxime	39.5	0	60.5
β-lactam (carbapenem)	Imipenem	97.4	0	2.6
Sulfonamides	Trimethoprim/sulfamethoxazole	44.8	13.1	42.1
Aminoglycosides	Streptomycin	39.5	23.7	36.8
	Gentamycin	71	10.6	18.4
Tetracycline	Doxycycline	42.1	31.6	26.3
	Tetracycline	39.6	15.7	44.7
Fluoroquinolones	Norfloxacin	71	8	21
	Levofloxacin	84.2	0	15.8
	Ciprofloxacin	68.5	5.2	26.3
Quinolones	Nalidixic acid	52.6	2.7	44.7
Phenicols	C30	86.9	0	13.1
Macrolide	Erythromycin	0	0	100
Nitrofurans	Nitrofurantoins	86.9	0	13.1

**Table-5:** Results of *bla*_TEM_*, bla*_SHV_*, bla*_CTX-M_*, bla*_OXA-1_*,* and *ampC* genes and antibiotic resistance pattern among *Escherichia coli* isolates.

Strain	Serotype	Source	Phenotypic antibiotic resistance	Resistance genes identified
Chicks				
1	O91	Chicks	P, E, CTX, SXT, S^10^, T^30^, NA^30^	*bla*_TEM_
2	O91	Chicks	P, E, SXT	
3	O91	Chicks	P, E, CTX, SXT	*bla*_CTX-M_
4	O91	Chicks	P, E, CF5	
5	O125	Chicks	P, E, SXT, S^10^, DO^30^, T^30^, NA^30^, F^300^	*bla*_SHV_
6	O122	Chicks	P, E, CTX, SXT, G^10^, T^30^, Lev, NA^30^	*bla*_TEM_*, bla*_SHV_*, ampC*
7	O145	Chicks	P, E, CTX, S^10^, DO^30^, C^30^	*bla*_SHV_*, ampC, bla*_CTX-M_
8	O145	Chicks	P, E, CTX	*bla*_SHV_*, bla*_CTX-M_
9	O78	Chicks	P, E, CTX, SXT, DO^30^, T^30^, NA^30^	*bla*_SHV_
10	O78	Chicks	P, E, CTX, SXT, DO^30^, T^30^, NA^30^	*bla*_CTX-M_
11	O44	Chicks	P, E, CTX, NA^30^	*bla*_CTX-M_
12	O36	Chicks	P, E	
13	UN	Chicks	P, E, NX^10^, CF5, IMP	*ampC*
14	UN	Chicks	P, E, CTX, SXT, S^10^, G^10^, T^30^, NX^10^, CF5, NA^30^, C^30^	*bla*_TEM_*, bla*_OXA-1_*, bla*_CTX-M_
15	UN	Chicks	P, E, CTX, SXT, S^10^, NX^10^, CF5, NA^30^, F^300^	*bla*_TEM_*, bla*_OXA-1_*, bla*_CTX-M_
16	UN	Chicks	P, E	*bla*_TEM_
17	O124	Chicks	P, E, CTX, G^10^, NA^30^	*bla*_TEM_
18	UN	Chicks	P, E, T^30^, C^30^	*bla*_TEM_
19	O26	Chicks	P, E, S^10^	*bla*_TEM_*, bla*_CTX-M_
20	O78	Chicks	P, E, S^10^	
Ducklings				
1	O91	Ducklings	P, NA, LEV, E, T^30^, S^10^, SXT, F^300^	*ampC*
2	O119	Ducklings	P, E, CTX, S^10^, T^30^, NA^30^, AMC	*bla*_TEM_*, ampC, bla*_CTX-M_
3	O145	Ducklings	P, E, S^10^	*ampC*
4	O36	Ducklings	P, E, NX^10^, NA^30^, CF5, CTX	*bla*_SHV_
5	O169	Ducklings	P, E, CTX, SXT, T^30^	*bla*_TEM_
6	O15	Ducklings	P, E, CTX, SXT, T^30^	*bla*_TEM_*, bla*_SHV_
7	UN	Ducklings	P, E, CTX	*bla*_TEM_*, bla*_OXA-1_*, ampC, bla*_CTX-M_
8	O15	Ducklings	P, E, CTX, SXT, S^10^, G^10^, T^30^, NX^10^, CF5, NA^30^, C^30^, F^300^	*bla*_TEM_*, bla*_CTX-M_
9	O15	Ducklings	P, E, C^30^	*bla*_TEM_*, bla*_OXA-1_
10	O15	Ducklings	P, E, S^10^, DO^30^, T^30^, NX^10^, CF5, F^300^	*bla*_TEM_*, bla*_SHV_*, bla*_OXA-1_*, bla*_CTX-M_
Turkeys				
1	O78	Turkey	P, E	
2	O28	Turkey	P, E, CTX, SXT, DO^30^, Lev, NA^30^, AMC	*bla*_TEM_*, ampC*
3	O28	Turkey	P, E, CTX, S^10^, DO^30^, T^30^, Lev, CF5, NA^30^	*bla*_TEM_*, bla*_SHV_*, ampC, bla*_CTX-M_
4	O29	Turkey	P, E, CTX, DO^30^	*bla*_TEM_*, ampC, bla*_CTX-M_
5	O168	Turkey	P, E, S^10^, G^10^, DO^30^, T^30^	
6	O125	Turkey	P, E, CTX, SXT, G^10^, DO^30^, T^30^, NX^10^, Lev, CF5, NA^30^	*bla*_TEM_*, bla*_SHV_*, ampC*
7	O158	Turkey	P, E, CTX, SXT, S^10^, G^10^, T^30^, NX^10^, Lev, CF5, NA^30^, F^300^	*bla*_SHV_*, bla*_CTX-M_
8	O115	Turkey	P, E, CTX	*bla*_TEM_

### Molecular detection of ESBL and ampC genes in E. coli strains

We identified one or more ESBL and/or *ampC* genes in 32/38 *E. coli* isolates (84.2%). *bla*_TEM_ was found in 20/38 (52.6%), *bla*_CTX-M_ in 15/38 (39.5%), *bla*_OXA-1_ in 5/38 (13.1%), and *bla*_SHV_, and *ampC* in 11/38 (28.9%) isolates each.

## Discussion

Although many studies have been conducted on the prevalence of *E. coli* in 1-day-old flocks, limited data were found on the prevalence of *E. coli* in imported 1-day-old flocks. *E. coli* is one of the most commonly spread bacterial pathogens in poultry worldwide and is responsible for colibacillosis, which usually presents as either a localized or systemic infection, leading to body weight loss and the contamination of a considerable number of carcasses during slaughter [21].

In our study, the prevalence of *E. coli* in 1-day-old imported flocks was 25.6%, which was slightly higher than the prevalence that was recorded in Dutch farms in 2-day-old grandparent stock chickens of broiler Breed A (23%) [22], but lower than prevalence rates reported in imported baby chicks in Egypt (44%) [23], 1-day-old chick Breed B in Dutch farms (44%) [22], and 1-day-old domestic and imported chicks in Egypt (60%) [24].

The prevalence of *E. coli* in imported duckling flocks in our study was 15.8%, which was higher than previously recorded in ducklings in Egypt (11.3%) [25]. In our study, the incidence of *E. coli* was 44% in imported chick flocks which are considerably higher than the incidence mentioned in 1-day-old chicks in Egypt (24% and 23.6% in Roshdy *et al*. [26] and Abdelrahman *et al*. [27], respectively). In the existing literature, the prevalence of *E. coli* in turkey poults flocks was 20%, which was lower than that reported in 1-day-old poults in Egypt (30% in 1-day-old) [28] but higher than the rate of another study (15%) conducted in Egypt [29]. In our study, 13.1% of *E. coli* isolates serotyped as O91, while in another Egyptian study, O91 was found in 5.8% and 4% of *E. coli* isolates from chicken and 1-day-old ducklings, respectively [26]. On the other hand, *E. coli* serotype O15 was identified in 10.5%, in our study, while O15 was detected in 4.1% of 1-day-old domestic and imported chicks in Egypt [24].

Multidrug resistance may emerge from the transmission of *E. coli* during hatching, breeding, and movement from one country to another, and the importation of poultry may also be regarded as another route of transmission of ESBL and *ampC* genes [22]. The development of the resistance to several antibiotics, such as gentamycin, SXT, ciprofloxacin, and cefotaxime, occurs due to their uncontrolled usage in treatment, or as growth promoters or prophylactics [30,31].

The global issue of antimicrobial resistance is steadily worsening. In this study, we examined the antimicrobial resistance of 38 *E. coli* isolates from imported poultry and found variable resistance rates. The high rates of resistance to tetracycline, SXT, and streptomycin as well as the low rates of resistance to gentamycin, norfloxacin, and ciprofloxacin, in our study, were in agreement with the results of a previous report from 1-day-old chicks in Egypt [24]. The rates of the antimicrobial resistance of isolated *E. coli* for amoxicillin-clavulanic acid, gentamycin, tetracycline, doxycycline, norfloxacin, and ciprofloxacin, in our study, were lower than those of *E. coli* isolates from turkey poults in Egypt [29] and higher than what was reported in a previous Egyptian study [24].

In Sweden, many cases of *E. coli* carrying ESBL or *ampC* resistance genes were recorded from the intestinal contents of broilers, and this was interpreted mainly as a consequence of the introduction of imported breeding stock in addition to antimicrobial use [32]. In addition, the global spread of MDR strains has caused a serious problem due to massive uncontrolled and random use of antimicrobials. In our study, 57.8% of *E. coli i* solates were MDR due to the exhibited resistance to at least one antimicrobial agent from three or more different antimicrobial classes [33]. In Egypt, two studies on *E. coli* isolates from turkey poults and day-old hatchlings showed that 100% [29] and 63.3% [34] were MDR, respectively. Collectively, these results are indicators of the frequent occurrence of MDR in the poultry industry.

Shortly after the release of the first extended-spectrum b-lactam antibiotics, bacterial resistance to such antibiotics began to emerge, and it has been reported as frequently increasing. The resultant therapeutic failures have turned into a worldwide problem [35]. In Europe, *bla*_CTX-M_, *bla*_TEM_, and *bla*_SHV_ have been recorded as the most predominant ESBL genes in compared with *ampC* [36]. Reports discussing the prevalence of ESBL-producing *E. coli* isolates from imported 1-day-old flocks in Egypt are scarce. Here, the *bla*_TEM_, *bl*a_SHV_, and *bla*_OXA-1_ genes were detected in 52.6%, 28.9 %, and 13.1%, respectively. Other studies have reported the aforementioned genes in 93.3%, 39.5%, and 60.0%, respectively, in *E. coli* isolates from 1-day-old hatchlings in Egypt [34]. In our study, *bla*_TEM_ and *bla*_OXA-1_ genes were detected in 52.6% and 13.1% of all *E. coli* isolates, respectively. Another study showed a very similar rate of the detection of *bla*_TEM_ and *bla*_OXA-1_ genes (52.3% and 14.3%, respectively) in *E. coli* isolates from poultry and beef products in Egypt [37].

The antimicrobial resistance is a serious global health problem among humans and in the field of veterinary medicine, and the existence of *bla*_CTX-M_ may cause the transfer of antibiotic resistance from poultry to humans through food supply chains or through mobile genetic elements in the surrounding environment [38]. *bla*_CTX-M_ was detected in 39.5% of *E. coli* isolates in this study. Furthermore, the β-lactamase-encoding genes *bla*_CTX-M1_ and *bla*_CTXM-2_ were detected in *E. coli* isolates from parent stock chickens of broiler Breed A in Dutch farms and in production farms and environmental samples of hatchery units at the broiler hatchery [22]. ESBL – as well as *ampC* – producing bacteria are commonly found in the gastrointestinal tract of animals [39] and have been isolated from turkey [40,41] and poultry [42]. The gastrointestinal tract of animals is seen as an important reservoir for bacteria that produce β-lactamases and as a potential source for human pathogens to take up these resistance genes [39,41,43]. ESBL and *ampC* genes that are located on plasmids are able to spread very rapidly [44]. The prevalence of *ampC* in both 1-day-old parent stock and 1-day-old broilers that were imported into the UK Dutch farms was 5.8% and 1.9%, respectively, which is markedly less that of our current study (28.9%) [22].

## Conclusion

The results presented in this study reveal the high prevalence of *E. coli* among imported 1-day-old poultry batches. Increasing resistance to several antibiotics was also detected, such as the resistance to cefotaxime and tetracycline, which may transfer such antibiotic resistance to humans. The presence of β-lactamase and *ampC* genes in the food chains of humans may also contribute to the problem of antibiotic resistance, which will directly result in public health problems.

## Recommendations

Surveys on the incidence and epidemiology of ESBL-producing *E. coli* in imported 1-day-old broilers shall be implemented and monitored. Furthermore, the controlled use of antibiotics is required to avoid the occurrence of antibiotic resistance to other antimicrobial drugs.

## Authors’ Contributions

MAAA, HR, and EAH designed the study, collected the samples, and applied bacteriological examinations. MA wrote the manuscript. AHS applied PCR testing. All authors have read and approved the final manuscript.
